# Congenital Cytomegalovirus Infection and Tetralogy of Fallot: An Unusual Association in a Three-month-old Baby

**DOI:** 10.7759/cureus.4949

**Published:** 2019-06-20

**Authors:** Nida Manzoor, Aiman Rehan, Manahil Akmal, Tayram B Khalid, Ammarah Jamal

**Affiliations:** 1 Pediatrics, Civil Hospital Karachi, Karachi, PAK; 2 Internal Medicine, Dow University of Health Sciences, Karachi, PAK

**Keywords:** cytomegalovirus, tetralogy of fallot

## Abstract

Congenital cytomegalovirus (cCMV) infection is the leading cause of infant morbidity and mortality worldwide. Despite being associated with significant neurological sequelae in infected infants, it remains an under-recognized public health entity. Symptomatic newborns most frequently display hepatosplenomegaly, petechiae, jaundice, microcephaly, intrauterine growth restriction, chorioretinitis, purpura, and seizures. Progressive sensorineural hearing loss is the most prominent adverse outcome of both symptomatic and asymptomatic CMV infections in infants. We report the case of a three-month-old baby who presented with complaints of progressive jaundice for three months and a two days history of fever associated with one episode of fits. The baby was diagnosed with congenital CMV infection on the basis of positive CMV IgM and IgG and positive maternal serum CMV IgG. Finding a murmur on physical examination prompted echocardiography which revealed Tetralogy of Fallot (TOF). The child was managed with a 6-week course of ganciclovir after which his symptoms improved and he was referred to cardiology for the evaluation of his heart defect. Follow-ups at the clinic have shown normal growth and development. This is the first reported association of cCMV infection with TOF. This case highlights the need to consider the possibility of the presence of heart defects in all infants with cCMV infection in addition to neurodevelopmental abnormalities. Clinicians should maintain a high degree of suspicion for cCMV infection in all neonates to ensure timely intervention and to prevent long-term neurological sequelae.

## Introduction

Congenital Cytomegalovirus (cCMV) infection is the leading cause of infant morbidity and mortality worldwide [1]. Despite being associated with significant neurological sequelae in infected infants, it remains an under-recognized public health entity [2]. It results from transplacental transmission of the virus either as a consequence of primary maternal infection or reactivation of the latent infection [3]. Permanent sequelae of cCMV can occur in any trimester but, infection during early pregnancy particularly during the period of organogenesis can be substantially more harmful [4]. Only ten percent of the infected infants are symptomatic at birth although long-term neurodevelopmental abnormalities have been observed even in otherwise asymptomatic infants [5]. Symptomatic newborns most frequently display hepatosplenomegaly, petechiae, and jaundice. Less common findings include microcephaly, intrauterine growth restriction, chorioretinitis, purpura, and seizures [5]. Progressive sensorineural hearing loss is the most prominent adverse outcome of both symptomatic and asymptomatic CMV infections in infants [6]. Although neurological manifestations of CMV infection are well recognized, it has rarely been reported in conjunction with congenital heart defects (CHDs). Reported cardiac abnormalities include aortic dilatation, ventricular septal defects (VSDs), and hypertrophic cardiomyopathies. Even in those cases, it was not clear whether such defects were directly caused by cCMV infection or were an incidental co-existence [7].

Tetralogy of Fallot (TOF) is the most common cyanotic CHD in children characterized by four cardiac defects: right ventricular enlargement, ventricular septal defect, overriding of the aorta, and right ventricular outflow tract obstruction. It has been described in association with chromosomal aberrations, maternal retinoic acid intake, uncontrolled diabetes mellitus, and phenylketonuria [8]. However, no association of cCMV infection with TOF has been reported in the literature so far. We report, for the first time, a case of cCMV infection in a three-month-old male baby with co-existent TOF.

## Case presentation

A three-month-old male baby, born of consanguinity, was brought to the Pediatrics Department of Civil Hospital, Karachi with complaints of jaundice, fever, and fits. Jaundice had first presented three months ago, on the third day of life, and was progressive. There was no history of fever, fits, vomiting, dark urine or pale stools during those three months. For the past two days, the baby had developed an intermittent, undocumented fever that was associated with one episode of generalized tonic-clonic fits lasting for ten minutes and followed by unconsciousness.

The patient’s mother had been an unbooked case in the prenatal period with no routine visits or tests done. She had an uneventful and normal vaginal delivery at term, carried out by a midwife at home. The baby cried immediately after birth but developed jaundice from the third day of life, which the parents had been trying to treat with home remedies. There were no feeding difficulties present and the family history was non-contributory. The baby had not been vaccinated according to the Expanded Program on Immunization (EPI).

On examination, the child was found to be jaundiced with microcephaly and impaired neuromotor development, exhibiting only partial control of his head and neck. He had started to recognize his mother for the past 20 days and was attracted by noise. Anthropometric measurements revealed that the patient’s weight was 4.5 kilograms and length was 55 centimeters, both below the third centile. The head circumference was 37.5 centimeters, also less than the third centile. On abdominal examination, hepatosplenomegaly was present and cardiovascular examination revealed a grade 2 pan-systolic murmur with signs of cyanosis evident during the examination.

**Figure 1:**
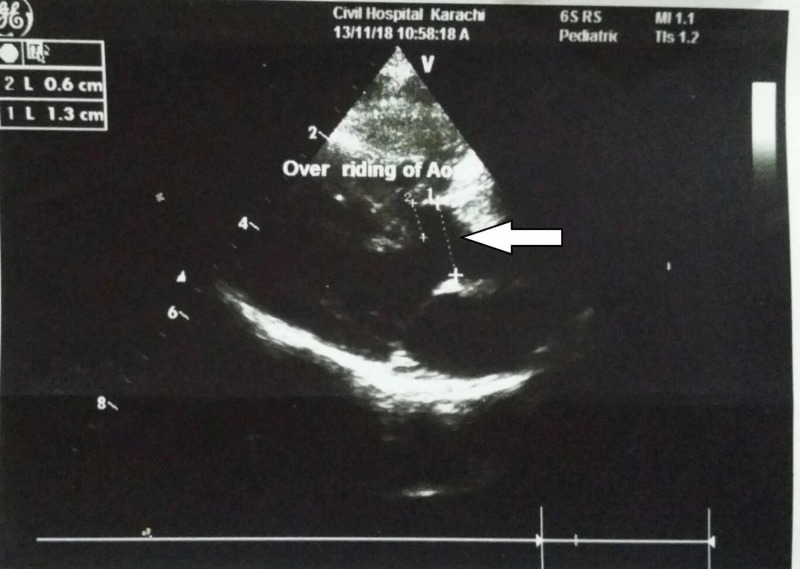
Transthoracic echocardiogram showing overriding of aorta

**Figure 2:**
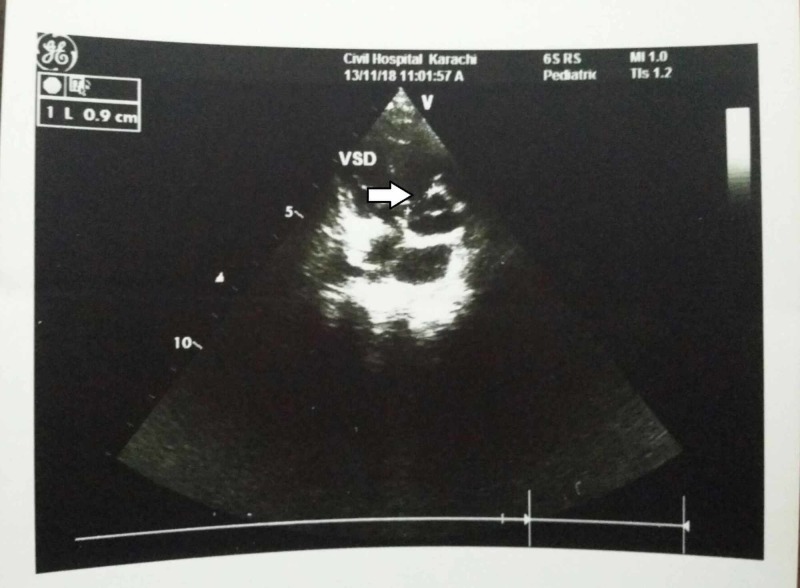
Transthoracic echocardiogram showing the ventricular septal defect

**Figure 3:**
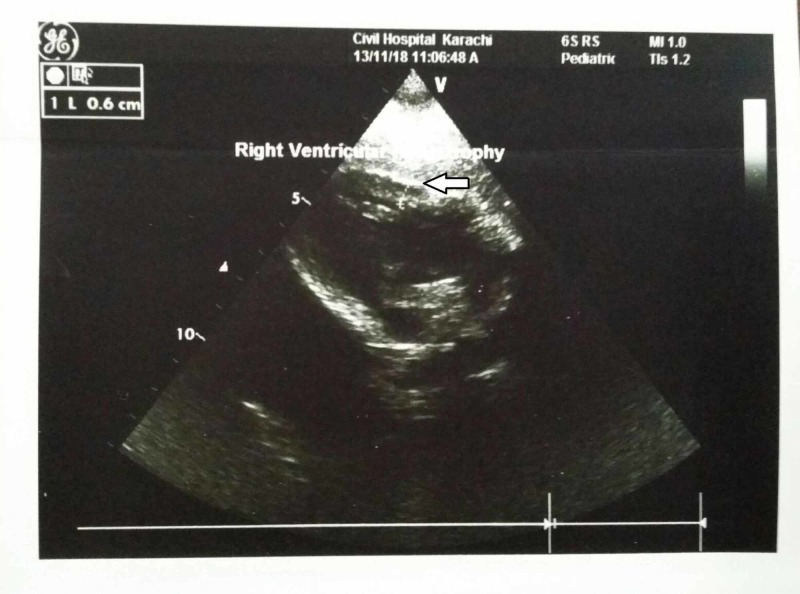
Transthoracic echocardiogram showing right ventricular hypertrophy

**Figure 4:**
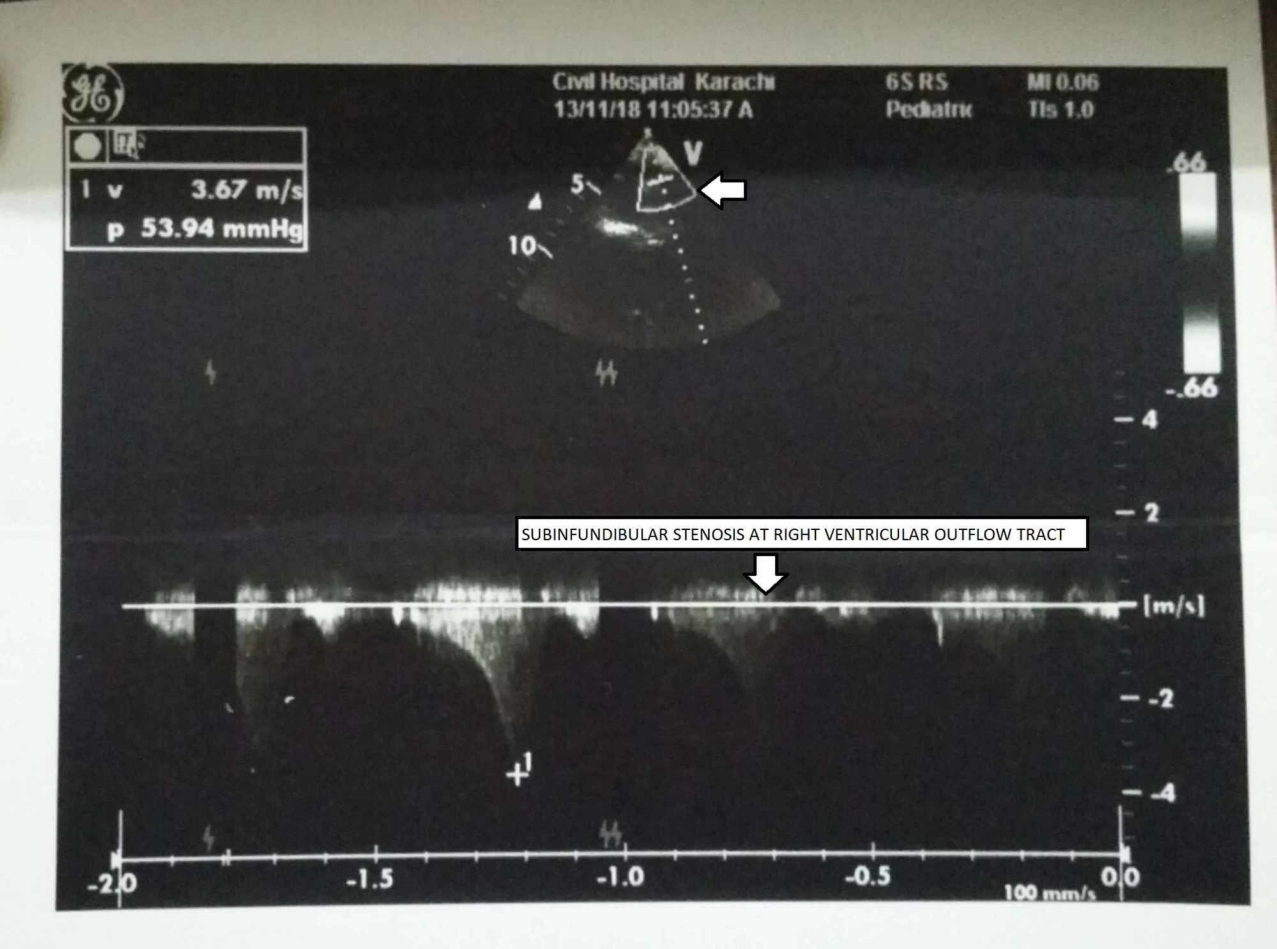
Transthoracic echocardiogram showing sub-infundibular stenosis at the right ventricular outflow tract

Basic laboratory investigations showed an elevated white blood cell (WBC) and platelet count, increased total and direct bilirubin and raised alanine aminotransferase (ALT) and aspartate aminotransferase (AST) levels. cCMV infection, biliary atresia, hypothyroidism, and rubella infection were among the differential diagnosis. Biliary atresia was ruled out when the ultrasound abdomen demonstrated a gallbladder. The thyroid stimulating harmone levels also came out to be normal, excluding hypothyroidism and the rubella testing was negative. Serum CMV IgM and IgG both came out to be positive, and the CMV DNA PCR was 20000 IU/ml. The mother’s CMV IgG was also positive indicative of the vertical transmission of the infection to the child during pregnancy. Further investigations revealed a TOF defect on echocardiogram (Figure 1-4), hepatosplenomegaly on ultrasound and bilateral moderate sensorineural hearing loss (SNHL) on brainstem evoked response audiometry (BERA) testing. The eye examination was normal, with no signs of chorioretinitis. Brain tomography was also normal, with no evidence of calcifications. The clinical and laboratory findings were highly suggestive of congenital CMV infection.

The patient was managed conservatively in the ward before the diagnosis of CMV was made after which he was started on Ganciclovir 10mg/kg/day, which was continued for six weeks. He had no subsequent episodes of fever or fits and his jaundice started resolving. During the course of his treatment, he was closely monitored for the development of neutropenia, the most commonly reported side effect of ganciclovir usage. The patient was discharged home after completion of the antiviral therapy. For his prevailing cardiac defect, he was referred to the Cardiology department. The surgery for TOF has been deferred until he is one year old. Meanwhile, to prevent the cyanotic spells due to his heart defect, he is being given oral propranolol 1mg/kg/6 hourly/day. Regular follow-ups in the clinics of the child have shown normal growth and development with all the milestones being achieved at a normal age. Repeat BERA testing has revealed no further deterioration in hearing.

## Discussion

Congenital CMV infection is a leading cause of infant morbidity and mortality across the globe. With an estimated prevalence of 0.2%-2.5%, it is the most common congenital infection and yet remains an underrecognized public health entity. Most of the infected infants are asymptomatic at the time of birth and only 10%-15% of them display symptoms such as hepatosplenomegaly, microcephaly, jaundice petechiae, and neurologic abnormalities. Hearing loss is the most adverse outcome of cCMV infection in both symptomatic and asymptomatic children [9].

Our patient presented with complaints of jaundice, fever, and fits. The presence of hepatosplenomegaly, microcephaly and retarded growth as indicated by reduced anthropometric measurements in our case are all compatible with the literature [10-11]. Seizures, although not very common, have also been reported as a manifestation of cCMV infection [11]. Absence of chorioretinitis in our patient is similar to a case reported by Dr. Khan [10], but unlike his report, our patient showed no evidence of calcification on brain tomography. BERA testing revealed bilateral moderate SNHL in our patient, although clinically he was responding to noise. Hearing loss can be a devastating consequence of cCMV with 9% of cases of all cases of SNHL attributed to CMV [12]. The current complications of cCMV themselves are a huge source of concern as they can be lethal and leave those who survive with permanent disabilities like deafness, blindness, and mental retardation [13] and the addition of the possibility of an increased risk of congenital cardiac anomalies with concurrent cCMV emphasizes the need to research the teratogenic effects of CMV on the fetal heart. 

Only a few cardiac abnormalities have been previously documented in association with cCMV. Dr. Khan reports two incidences of aortic dilation which were associated with cCMV and resolved on treatment [10]. A case of cardiac inflammation was reported post mortem by C. P. Barnett, et al [14] when abnormalities were seen on echocardiography and the couple chose for termination. However, no case of TOF has been reported in conjunction with cCMV. The exact mechanisms by which viruses may cause CHD remains elusive; vascular compromise as a result of endothelial cell damage and disruption in DNA replication are implicated. Although much of the evidence in this regard comes from congenital rubella syndrome [7], similar mechanisms could be responsible for the heart defects seen in cCMV. Evidence suggests that timely administration of antiviral therapy halts the progression of hearing impairment in neonates [15]. The child in our case also seemed to have benefited from the antiviral treatment as repeat BERA testing has revealed no further deterioration in hearing. His normal growth and development are also indicative of a favorable outcome as a result of suitable treatment. To monitor the development of any further neurological deficits, he is being regularly followed up.

Prevention from CMV is the mainstay of avoidance of CMV-related congenital anomalies. Vaccination has been a success in, if not completely erasing but significantly reducing incidences of congenital rubella, poliomyelitis, and H. influenza meningitis while educating mothers on not consuming alcohol has reduced fetal alcohol syndrome and folic acid intake has reduced neural tube defects [15]. But when it comes to CMV, there is a lack of both vaccination and education. CMV, like other herpes viruses, presents as primary infection and then remains in the body for a life-long latent phase with a potential of reactivating at any point. CMV is present in all bodily fluids and is transmitted by close personal contact of both sexual and non-sexual nature, breastfeeding, and blood transfusions [15]. Pregnant women, however, are most likely infected by coming in contact with urine and saliva of other children. A number of clinical trials are undergoing an assessment with a vaccine against the glycoprotein B showing a 50% efficacy to prevent infection [16-17]. However, until an effective vaccine becomes available, the mainstay of focus should be educating pregnant women on ways to avoid this lethal infection [13,18-19].

## Conclusions

Our case is the first reported association of TOF with congenital CMV. While the causative role of CMV in congenital heart defects cannot be firmly established on the basis of this case, it highlights the need to consider the possibility of the presence of heart defects in all infants with congenital CMV infection in addition to neurodevelopmental abnormalities. Clinicians should maintain a high degree of suspicion for cCMV infection to ensure timely intervention and to prevent long-term neurological sequelae. There is also a dire need of clinical research focused on discovering the teratogenic effects of CMV on the heart and other organs. Since most infants are asymptomatic at birth, the possibility of the implementation of a newborn screening program should also be explored to protect against long-term neuordevelopmental abnormalities that may arise in otherwise asymptomatic children later in life.
